# Mechanism of nutrition activity of a microgranule fertilizer fortified with proteins

**DOI:** 10.1186/s12870-020-02340-4

**Published:** 2020-03-24

**Authors:** Maksymilian Olbrycht, Michał Kołodziej, Roman Bochenek, Mateusz Przywara, Maciej Balawejder, Natalia Matłok, Piotr Antos, Wojciech Piątkowski, Dorota Antos

**Affiliations:** 1 Department of Chemical and Process Engineering, Rzeszow University of Technology, Powstańców Warszawy Ave. 6, 35-959 Rzeszów, Poland; 2 Department of Chemistry and Food Toxicology, University of Rzeszow, St. Ćwiklińskiej 1a, 35-601 Rzeszów, Poland; 3 Department of Food and Agriculture Production Engineering, University of Rzeszow, St. Zelwerowicza 4, 35-601 Rzeszów, Poland; 4 Department of Computer Engineering in Management, Rzeszow University of Technology, Powstańców Warszawy Ave. 10, 35-959 Rzeszów, Poland

**Keywords:** Diffusion in soil, Maize fertilization, Protein fortified fertilizer

## Abstract

**Background:**

A microgranule fertilizer was designed for localized fertilization of soil with controlled release of nutrients. The microgranule matrix was fortified with proteins, which were obtained from food industry byproducts or waste, i.e., whey protein from milk serum, soy protein from soy isolate and egg white protein from chicken egg white powder. The mechanism of the protein decomposition and migration of micro and macromolecule compounds through two different model soil systems was investigated. The potential of the protein fortified fertilizer for localized fertilization of the potted maize seeds was evaluated.

**Results:**

The study revealed that proteins slowly diffused through soil with simultaneous degradation, which was accompanied with release of ammonia ions. The highest concentration of proteins and degradation products was found in a close vicinity of the microgranule. The microgranules were used as a local fertilizer for maize seeds in the pot experiments. The experiments confirmed statistically significant improvement in root density of maize plant compared to control group.

**Conclusions:**

Byproducts or waste of food industry, such as the milk serum and soy can be used as a source of proteins that degrade in soil without a pretreatment. The degradation is accompanied with formation of ammonium ions, which can be utilized by plants as a nitrogen source. The fertilizer microgranule should be placed in a close vicinity to the plant seed, since the maximum of the protein concentration and ammonia ions is reached at a very close distance from the microgranule.

## Background

Within the last decades various fortified fertilizers have been applied to stimulate yield of plant crops. Particular interest arose in biostimulating additives, including: humic substances, seaweed extracts and amino acids containing products [1–4].

Humic substances and seaweed extracts contain plant growth hormones, such as auxins and gibberellins. Since plants are affected by slight amounts of plant growth hormones, those substances have impact on plant growth even in low amounts [2, 3, 5–8].

Biostimulants based on amino acids are produced through chemical synthesis from plant proteins, such as algae, maize or soyabean [9]. Another source is chemical or enzymatic hydrolysis of animals or plant proteins [5, 10–16].

Protein hydrolysates mainly consist of peptides, oligopeptides and amino acids, but may also contain carbohydrates, small amounts of mineral elements, phenols and other compounds. Detailed chemical characteristics of protein hydrolysates depend on the origin of proteins (plant animal material).

Protein hydrolysates stimulate carbon and nitrogen metabolism and regulate the nitrogen uptake. Bioactive peptides that are contained in hydrolysates can have an impact on hormonal activities [5]. Moreover, peptides and amino acids coming from protein hydrolysates have the ability to form complexes and chelates with soil micronutrients (i.e., Cu, Fe, Mn and Zn), which contributes to the improved availability and acquisition of nutrients by the root system and enhances the density of the root system [2, 17–19].

The protein hydrolysates play also an important role as nutrients for microorganisms living in the rhizosphere and phyllosphere of plant organisms. Thus, they affect the interaction between plants and microorganisms present in soil [5]. Corte and co-workers analyzed different protein hydrolysates obtained by thermal, chemical and enzymatic hydrolysis and showed that they do not negatively affect soil ecosystems and that they can be used in farming as a nitrogen source [20].

A significant contribution of protein hydrolysates used either in soil or foliar treatments to gain high yields of plant crops was reported in several studies. For example, Marfa and co-workers applied enzymatic hydrolysates obtained from animal hemoglobin on strawberry plants in the initial growing stages [21]. The results they obtained indicated a significant increase in the early production of fruit compared to the control group. Ertani and co-workers, who utilized two different fertilizers based on alfalfa and hydrolyzed meat flour, proved that protein hydrolysates can not only have a positive impact on plant growth, but also on the condition of the environment since they increase fertilizer efficiency, thus reducing the amount of fertilizers needed in the agricultural production [22]. Colla and co-workers reported a positive effect of plant-derived protein hydrolysate on the hormone-like activity, nitrogen uptake and growth stimulation of maize, tomato and dwarf pea [23]. Subbarao and co-workers observed that the application of protein hydrolysate resulted in increase in both root growth and leaf area in crops of rice, finger millet, radish, and cowpea [16]. Liuciatelli and co-workers indicated that a protein hydrolysate-based biostimulant derived from a legume and another product derived from tropical plant extracts altered the composition of the plant growth-promoting microbial population in lettuce [24]. Popko and co-workers demonstrated the potential of biostimulation of plants by foliar application of preparations based on keratin hydrolysates obtained from feathers [15]. The authors reported an increase in the yield of plant crops when compared to a control group and indicated that the increased efficiency of the tested biostimulants arose from the presence of micro- and macronutrients in hydrolysates of keratin protein.

In this study, we developed a microgranule fertilizer fortified with a protein material, which was embedded into microgranules without chemical or enzymatic pretreatments. The purpose of the study was twofold. Firstly, we examined the mechanism of degradation of protein fortifiers in soil in situ and migration of nutrients through soil. Secondly, we aimed at evaluation of the potential of the protein fortified fertilizer to be used for localized soil fertilization. Localized application is as an attractive technique of fertilization that can surpass broadcast applications in economic, environmental and technological aspects. It can effectively be used for manipulating root morphology by stimulation of root proliferation and for improvement of the nutrient uptake [25].

In the study we used three different protein fortifiers, which were sourced from byproducts or wastes of food industry, i.e., milk serum, soy, and egg white powder. The protein material was embedded in a mineral phosphate matrix prepared in the form of a microgranule, which contained calcined and grounded bones obtained from meat industry waste. We employed different analytical methods to follow the progress of protein degradation in standardized soil samples, as well as migration of the microgranule components through two different soil systems. To verify the positive effect of the fertilizers on the plant growth, we performed preliminary tests for the application of the microgranules to localized fertilization of the potted maize seeds. We determined the effect of fertilization on both the root density and aboveground parts of the plant in the initial phase of the maize growth.

## Results

### Content of mobile ions in standardized soil systems

As reported above, two different types of standardized soil were used: sand (soil I), which was supposed to mimic soil with organic matter-poor content, and a mixture of peat and sand (soil II), which was meant as a model of soil with organic matter-rich content. To determine the content of mobile ions in both systems, blank samples of soil that was not contacted with microgranules, were subjected to extraction in deionized water, and the obtained extracts were subsequently analyzed by ion chromatography. The results of the analysis are presented in Table 1.

**Table 1:** Mean concentration of the ions extracted from control samples of soil I and II, in deionized water (μg per g of the soil) obtained in triplicated experiments (Soil I, Soil II); and the mean concentration of ions released into the aqueous environment from the granulate free of the protein (μg per g of granulate)

	Na^+^	K^+^	Mg^2+^	Ca^2+^	$\mathrm{N}{\mathrm{H}}_4^{+}$	Cl^−^	$\mathrm{N}{\mathrm{O}}_3^{-}$	$\mathrm{P}{\mathrm{O}}_4^{3-}$	$\mathrm{S}{\mathrm{O}}_4^{2-}$
soil I	3.5	2.9	2.3	16.4	1.7	2.2	0.9	–	106.7
soil II	7.7	14.2	3.1	75.4	12.6	3.2	15.9	–	149.1
microgranule free of the protein	3.8	1.5	0.1	0.2	0.0	0.1	0.1	0.5	2.9

Both model soils contained a high amount of sulfate anions $\mathrm{S}{\mathrm{O}}_4^{2-}$; soil II was also rich with Ca^2^^+^ ions. Furthermore, in both soils several different ions were detected in a smaller amount, whereas the amount of phosphate ions was below detectable level.

### Mobility of microgranule ions in water

To determine the mobility of ionic components from the fertilizer matrix in aqueous environment, the microgranule free of the protein fortifier was covered with deionized water. Samples of the supernatant were acquired and subjected to the concentration analysis by ion chromatography. The mean mass of ions released in triplicated experiments was normalized with respect to the mass of the microgranule. The results are presented in Table 1. It can be seen that several ions are released into the water environment: Na^+^, K^+^, $\mathrm{S}{\mathrm{O}}_4^{2-}$, with the highest amount (i.e., above 1 μg per g of microgranule), $\mathrm{P}{\mathrm{O}}_4^{3-}$ with the amount of about 0.5 μg per g microgranule and a smaller amount of Ca^2^^+^, Mg^2+^, $\mathrm{N}{\mathrm{O}}_3^{-}$, and Cl^−^. pH of the solution was 8.6 – which came from mobile hydroxyl ions of the matrix. This indicated an alkaline environment inside the matrix, which was expected to be favorable for gradual degradation of proteins.

### Mobility of microgranule components in soil I

#### Microgranule fortified with whey protein isolate

**Fig. 1:**
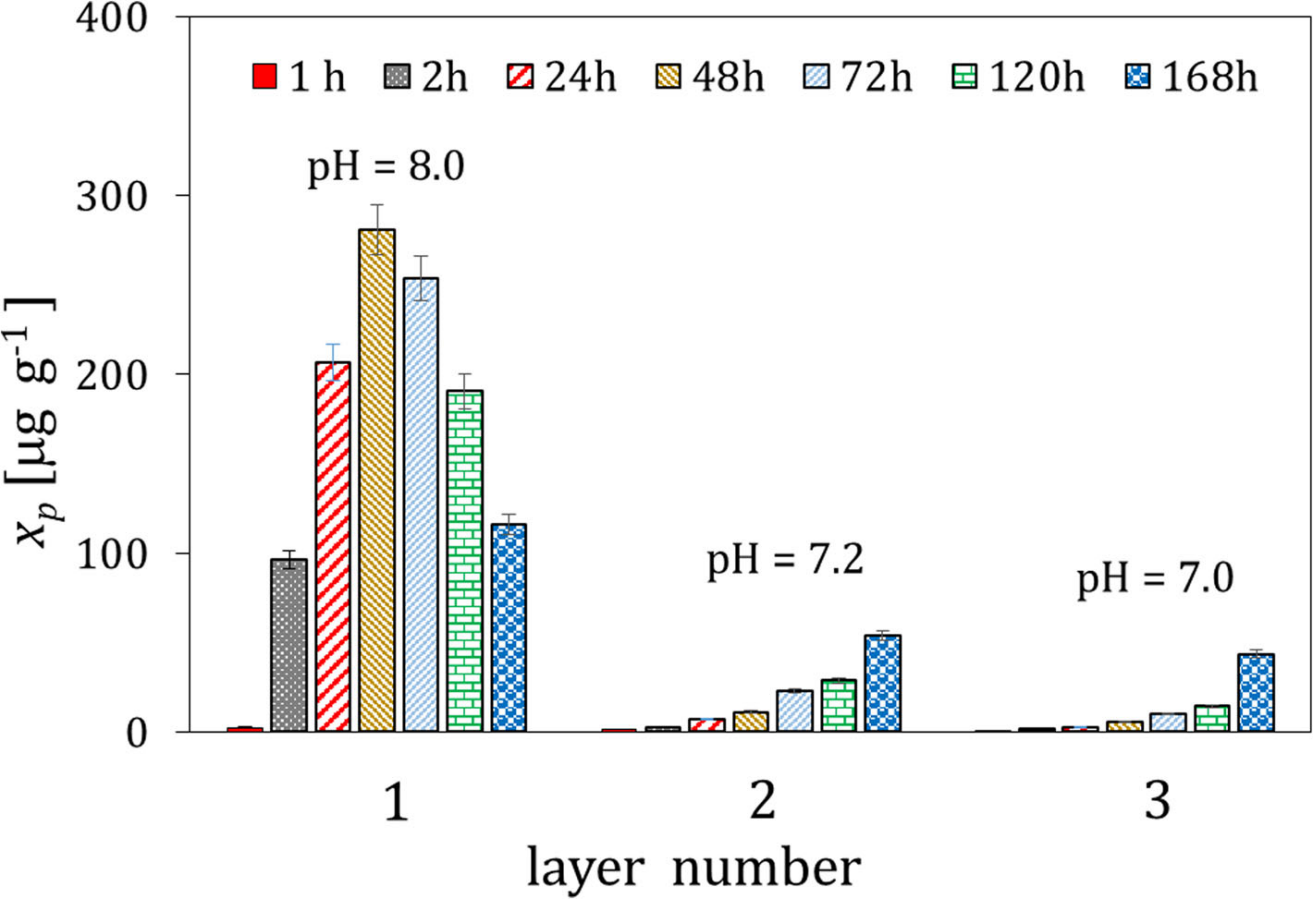
Changes in the mean protein concentration with time in the extracts from three soil layers acquired at different distances from the microgranule fortified with the whey protein isolate (soil layer 1: 0–1.5 cm, layer 2: 1.5–3 cm, layer 3: 3–4.5 cm)

**Fig. 2:**
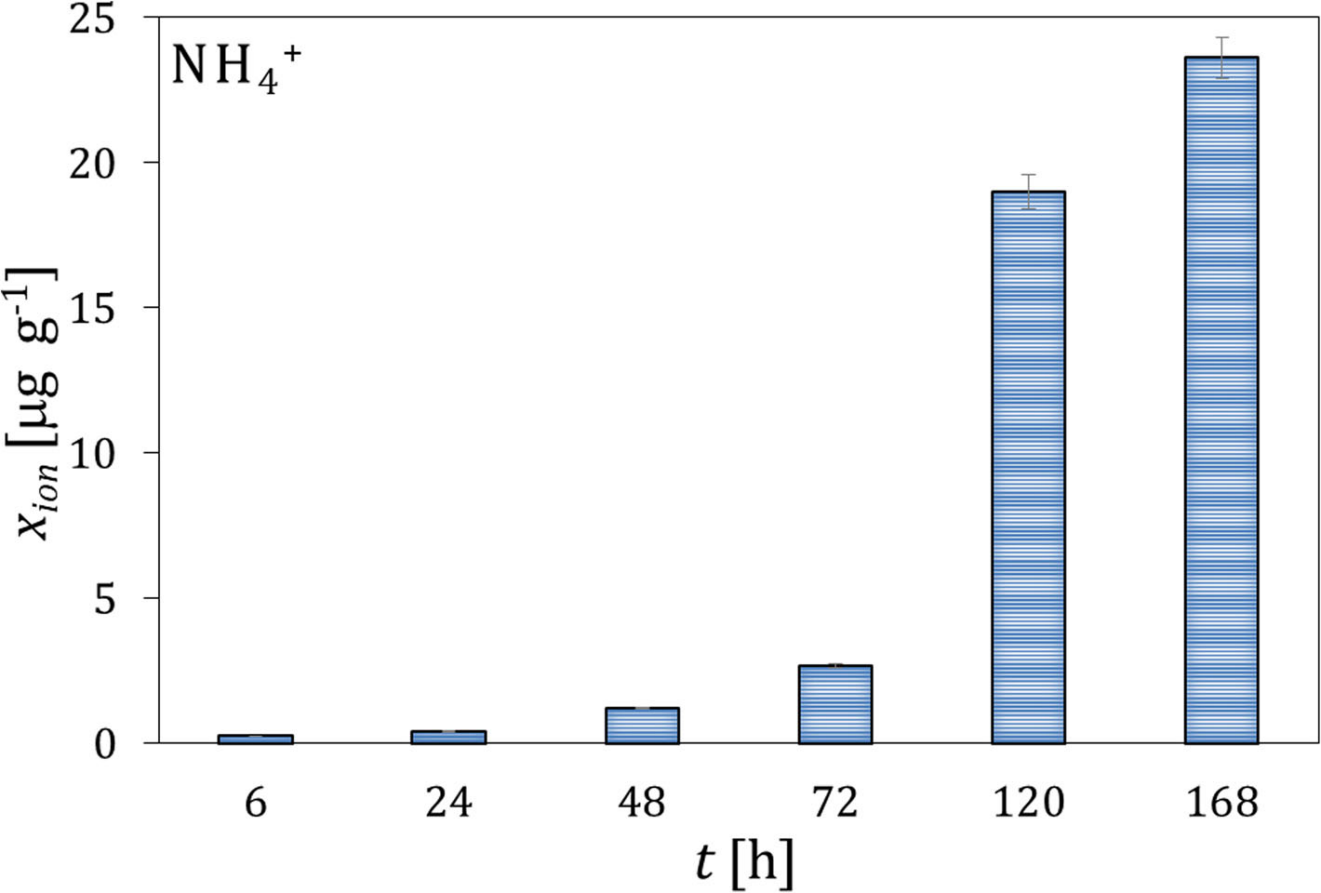
Changes in the mean excess concentration of $\mathrm{N}{\mathrm{H}}_4^{+}$ ions with time in the extracts from the soil layer 1 surrounding the microgranule fortified with the whey protein isolate; the concentration of ions in the blank soil sample (without the presence of the microgranule) was subtracted from the concentration measured in the presence of the microgranule

**Fig. 3:**
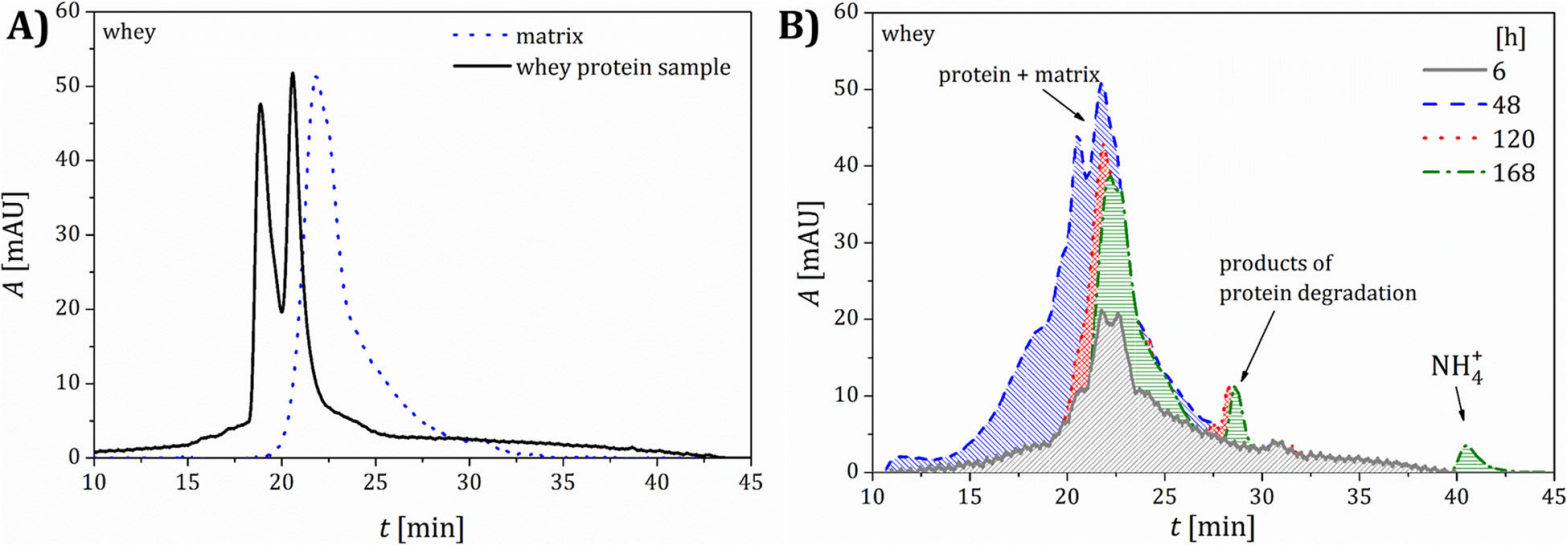
SEC-HPLC chromatograms for a standard solution and extracts of soil I. **a** Whey protein isolate as a standard and the extract from the soil layer 1 acquired after 72 h; **b** extracts from the soil layer 1 acquired at different time intervals

The microgranules of the fertilizers fortified with the whey protein isolates and free of the protein material were contacted with soil I. Typical results of the concentration analysis of the protein content by the Lowry method and pH in the extracts from the soil layers, which were acquired at different distances from the microgranule and different time intervals, are presented in Fig. 1. It can be observed that the maximum of the protein concentration is reached after 48 h in the soil layer 1, in direct vicinity of the microgranule. The concentration of the protein drops rapidly with increasing the distance from the microgranule, and vanished in the last layer 4 (not shown). Moreover, the concentration of the protein decreases in time, which is caused by its degradation. The latter is confirmed by increasing the $\mathrm{N}{\mathrm{H}}_4^{+}$ concentration with time, which is presented in Fig. 2, where the excess concentration of $\mathrm{N}{\mathrm{H}}_4^{+}$ ions in extracts of soil contacted with the microgranule is depicted. Additionally, changes in molecular weight of the protein fortifier in extracts of the soil layer 1, which were acquired at different time intervals, are illustrated on the SEC-HPLC chromatograms in Fig. 3.

The SEC-HPLC analysis confirms that the highest concentration of the protein is reached after 48 h, then the peak assigned to the protein gradually vanishes. For the samples acquired after 120 h, additional peaks appear on the rear part of the chromatogram, which can be assigned to small molecular products of the protein degradation and $\mathrm{N}{\mathrm{H}}_4^{+}$ ions.

**Fig. 4:**
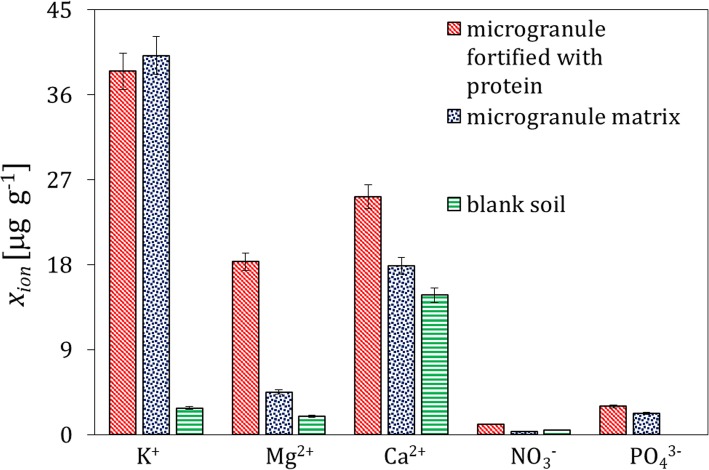
Mean concentration of ions in the extracts from the soil layer 1 acquired after 168 h; blank sample without the contact with the microgranule, sample after the contact with the microgranule free of the protein, and with the microgranule fortified with the whey protein isolate

The concentration of other ions in the soil extracts faster reaches its maximum (i.e., after 24 h) and spreads farther compared to the protein material, which stems from higher diffusivity of small ions in the solid matrix. An illustration of the migration rate of K^+^ is shown in Supplementary materials, Fig. S1. The concentration of ions in the extracts from the soil layer 1 contacted with the microgranule with and without the protein, and in the blank soil extracts is presented in Fig. 4. It can be observed that the protein isolate also appears as a source of Mg^2+^. Moreover, a small excess of Ca^2^^+^ ions is observed when compared to the microgranule without the protein. It is also apparent that the ions of K^+^ and $\mathrm{P}{\mathrm{O}}_4^{3-}$ are released from the microgranule matrix (i.e., from calcined bones), they are not present in the fortifiers. The effect of migration of the $\mathrm{S}{\mathrm{O}}_4^{2-}$ ions contained in the microgranule matrix is not shown since they were also present in bank soil samples (Table 1).

#### Microgranule fortified with soy protein isolate

**Fig. 5:**
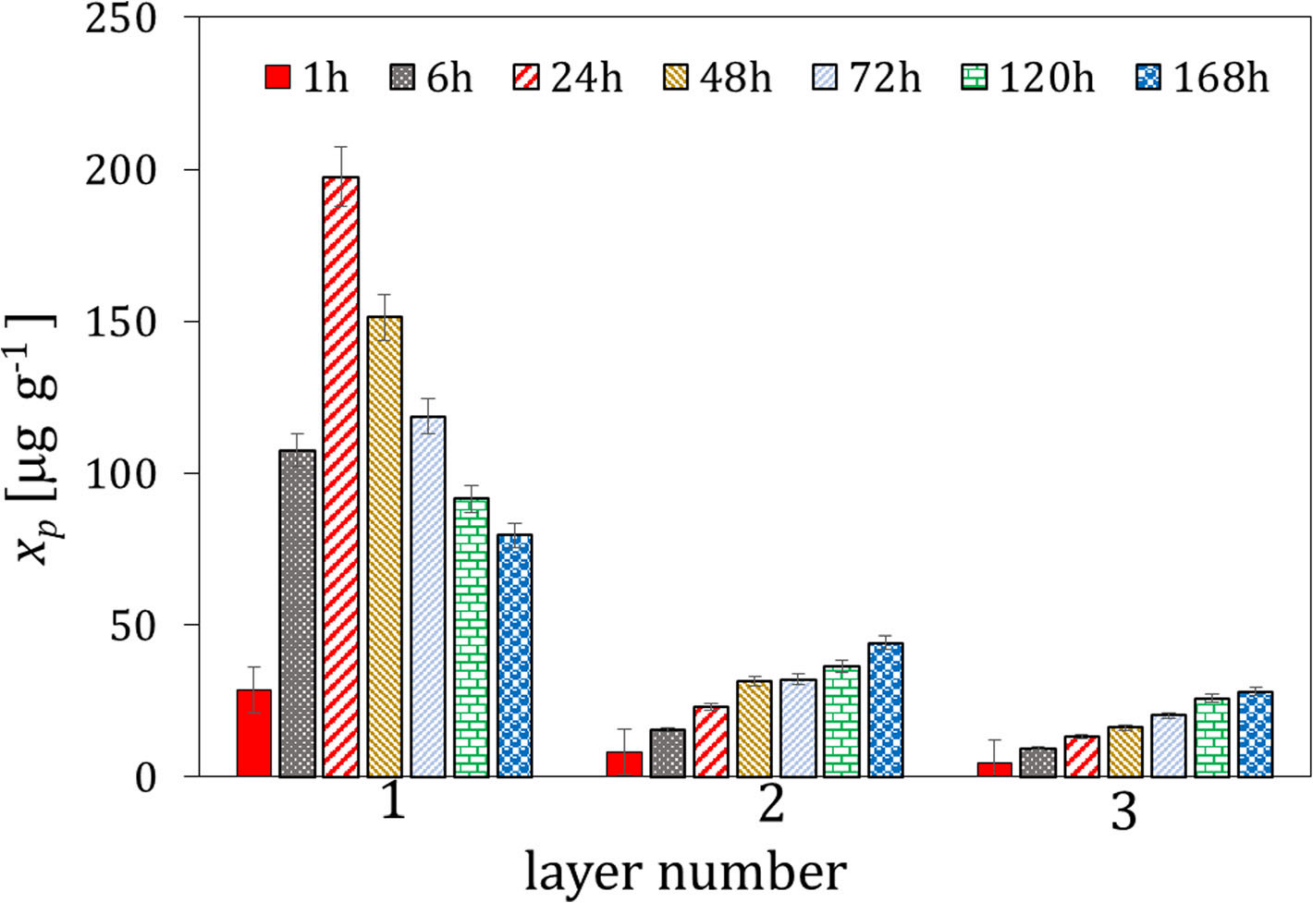
Changes in the mean protein concentration with time in the extracts from three soil layers acquired at different distances from the microgranule fortified with the soy protein isolate (compare to Fig. 2)

**Fig. 6:**
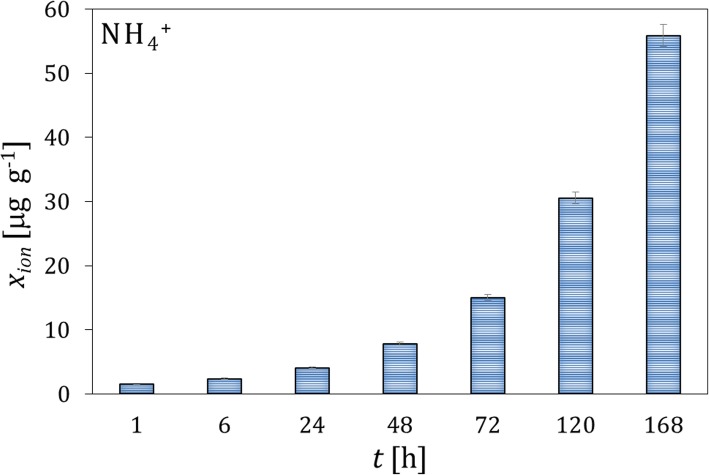
Changes in the mean excess concentration of $\mathrm{N}{\mathrm{H}}_4^{+}$ ions with time in the extracts from the soil layer 1 surrounding the microgranule fortified with the soy protein isolate (compare to Fig. 3)

**Fig. 7:**
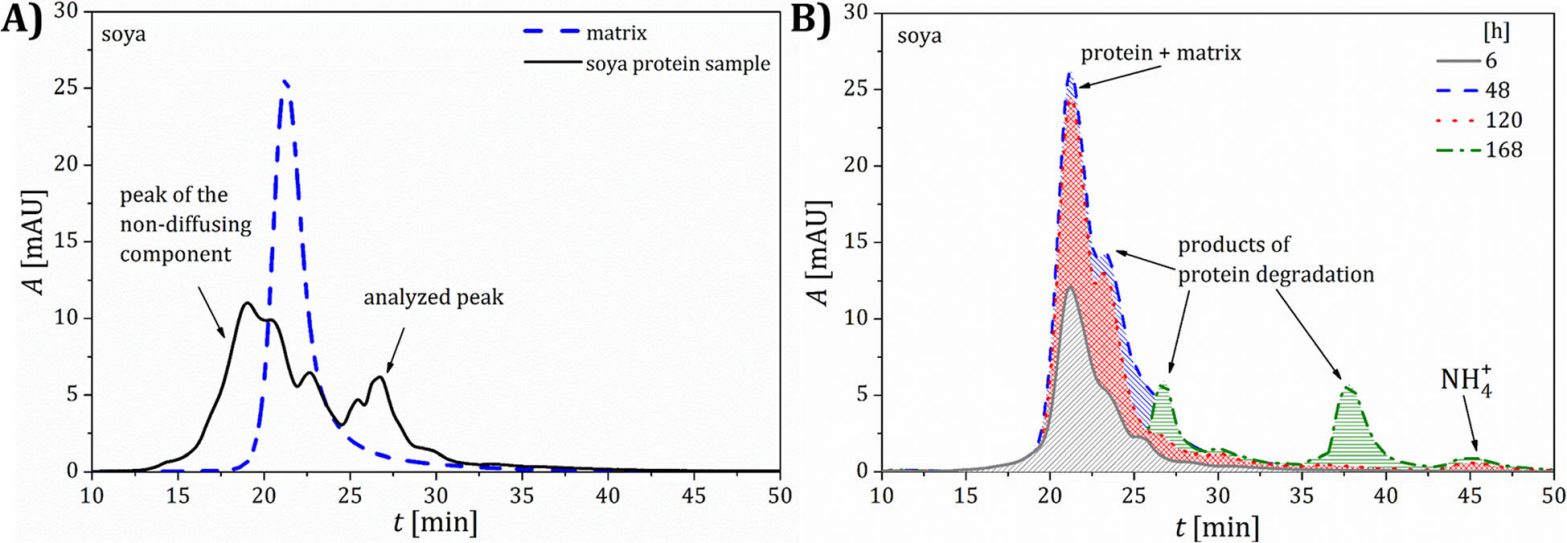
SEC-HPLC chromatograms for a standard solution and extracts of soil I. **a** Soy protein isolate as a standard and the extract from the soil layer 1 acquired after 72 h; **b** extracts from the soil layer 1 acquired at different time intervals

Similar experiments were performed with the microgranules fortified with the soy protein isolate. The results obtained are depicted in Figs. 5, 6 and 7. The maximum of the average protein concentration in extracts from the soil layer 1 is reached after 48 h (Fig. 5), similarly as it was observed for the microgranules with the whey protein isolate.

The changes in the $\mathrm{N}{\mathrm{H}}_4^{+}$ ion concentration with time are illustrated in Fig. 6. The amount of $\mathrm{N}{\mathrm{H}}_4^{+}$ ions released from the microgranules fortified with the soy protein isolate is higher compared to the microgranules with the whey protein isolate, with however slower kinetics of release. The substantial increase in the $\mathrm{N}{\mathrm{H}}_4^{+}$ concentration occurs at 168 h of measurements, whereas in case of the microgranule fortified with the whey protein isolate it corresponded to 120 h (compare Fig. 2 and Fig. 6).

The changes with time in molecular weight of the components of the extracts from the soil layer 1 surrounding the microgranule with the soy protein isolate are shown on the SEC-HPLC chromatograms in Fig. 7a, b.

The SEC-HPLC analysis reveals that the highest concentration of the protein is achieved after 48 h, later the peaks corresponding to the protein material disappear (Fig. 7b). A lack of peaks corresponding to large molecular weight components can be observed while compared to the protein isolate standards (Fig. 7a). This indicates that they do not diffuse through the soil within the time interval investigated. It can be also observed that molecular weight of the macromolecules in the soy protein isolate is higher compared to the whey protein (Fig. 3).

**Fig. 8:**
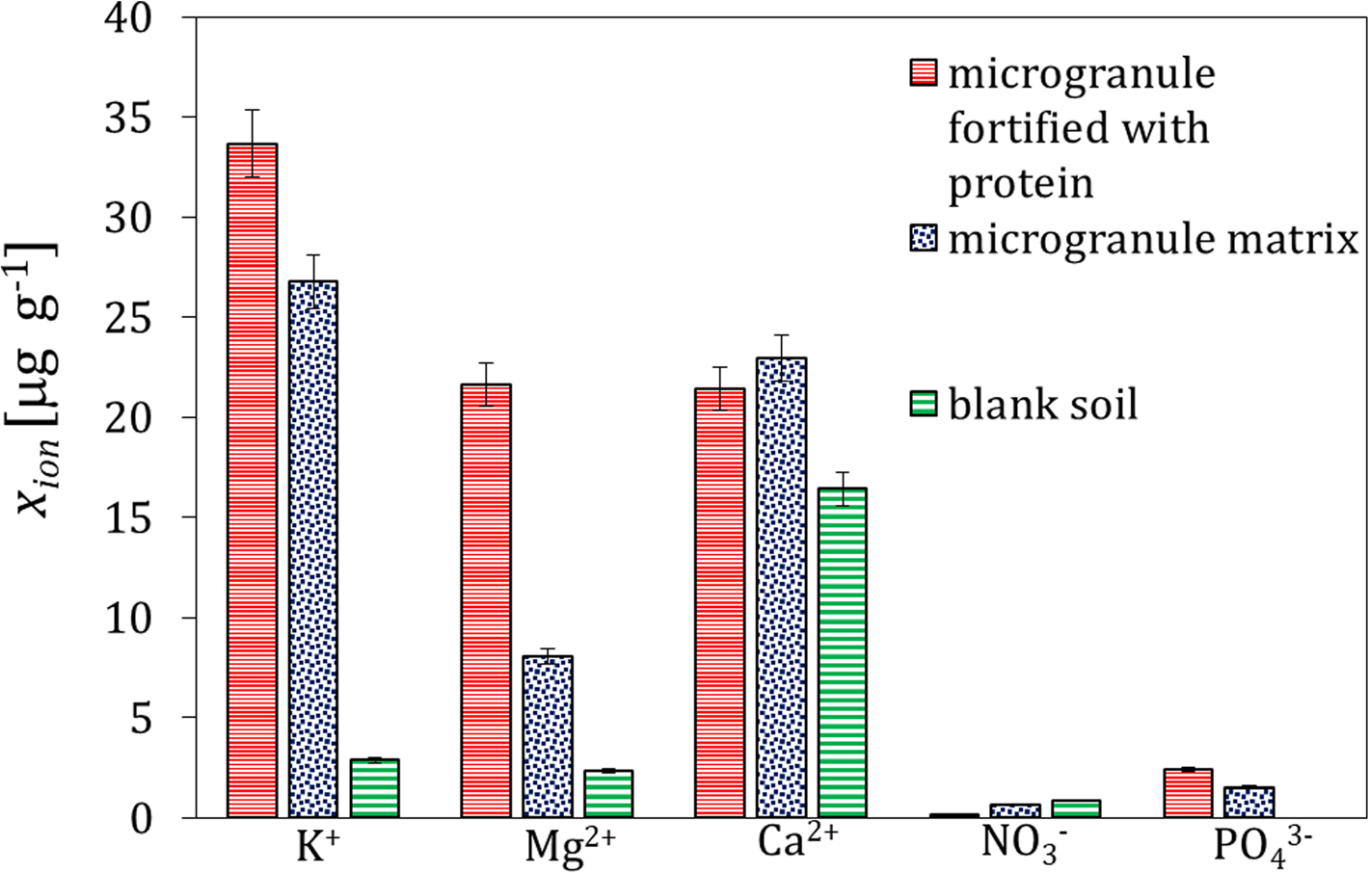
Mean concentration of ions in the extracts from the soil layer 1 acquired after 168 h; blank sample without the contact with the microgranule, sample after the contact with the microgranule free of the protein, and with the microgranule fortified with the soy protein isolate

The results of the ion analysis presented in Fig. 8 are very similar to that shown in Fig. 4 for the experiments with the whey protein-supplemented microgranules.

#### Microgranule fortified with egg white powder

Finally, the transport of the protein material through soil I was investigated for the microgranule fortified with egg white powder. In this case, the protein reached the soil layer 1 relatively quickly, faster than for the remaining fortifiers, but the protein amount did not decrease in time. This indicated that the protein did not degrade. It can be probably attributed to very low solubility of egg white powder in water. The phenomenon was confirmed by the $\mathrm{N}{\mathrm{H}}_4^{+}$ concentration analysis, which revealed that the excess concentration of $\mathrm{N}{\mathrm{H}}_4^{+}$ ions was very low. The SEC-HPLC analysis revealed relatively fast diffusion of protein in soil as well as lack of degradation of the protein over the time interval of 168 h (the protein peak area remained the same between 48 and 168 h).

The changes in the egg protein concentration in soil, the excess concentration of $\mathrm{N}{\mathrm{H}}_4^{+}$ ions and the results of the SEC-HPLC analysis are shown in Figs. S2, S3, S4 and S5 in Supplementary materials.

### Mobility of microgranule components in soil II

**Fig. 9:**
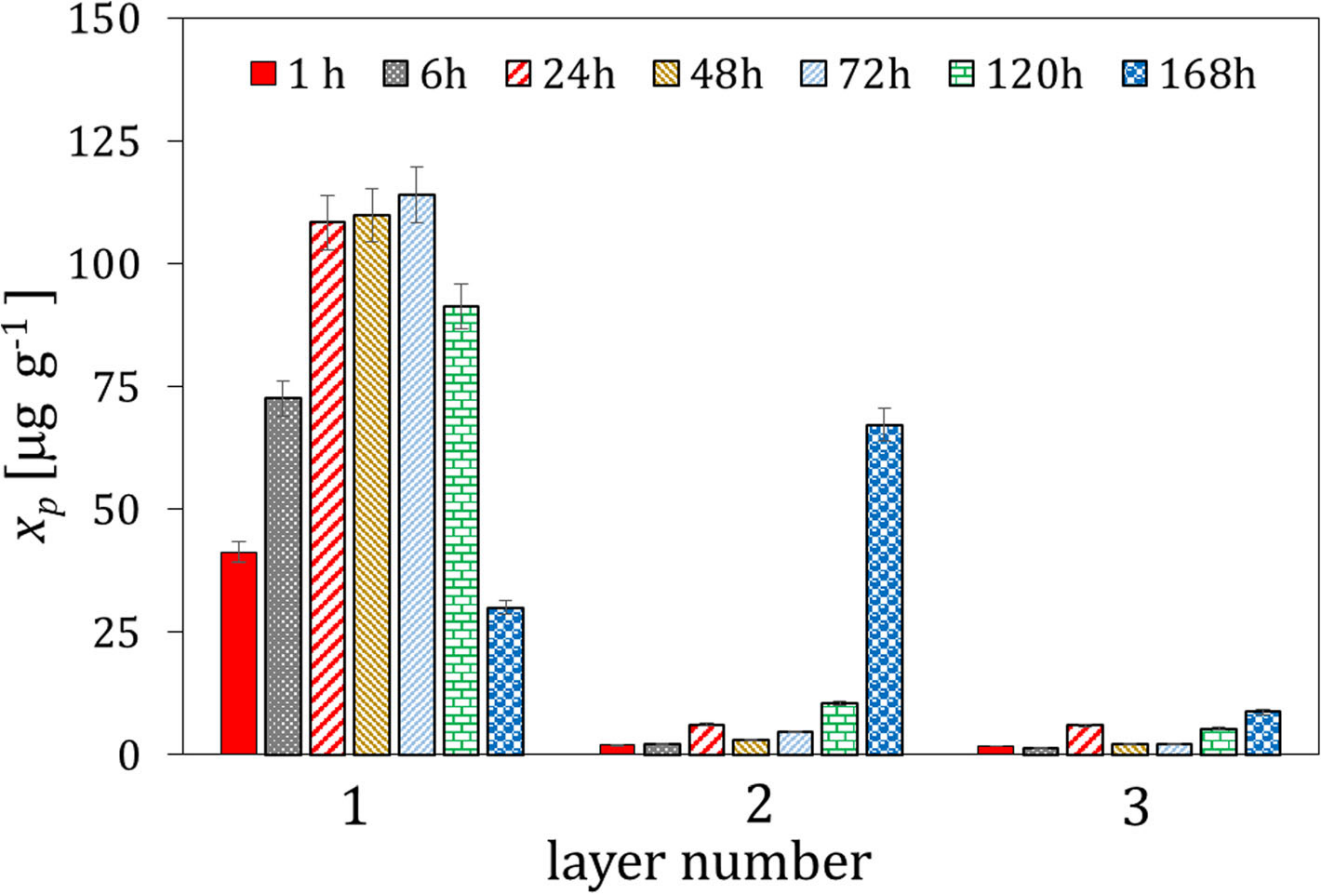
Changes in the mean protein concentration with time in extracts from three soil II layers acquired at different distances from the microgranule fortified with the whey protein isolate (compare to Fig. 2)

The experiments were also performed for soil II that was rich with organic matter. A typical illustration of transport of the whey protein through the soil is shown in Fig. 9. The distribution of the protein in the soil layers is similar to that reported for soil I, but the concentration of the protein is lower. This can be explained by the presence of the organic matter which might hinder diffusion of the protein, as well as stimulate microbial decomposition of the protein.

### Pot experiments of maize plant cultivation

**Fig. 10:**
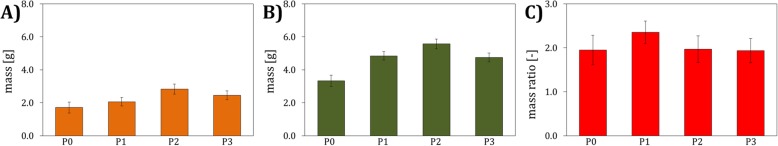
Dry mass for different test variants: **a**) of the root; **b**) of the aboveground part of the plant; **c**) mass ratio of the root to the aboveground part of the plant in 33 days after sowing (BBCH14)

The microgranules fortified with the whey and soy protein were used for localized fertilization for maize seeds. The microgranule was placed at a 2 cm distance from the seed. That distance was selected on the basis of the performed analysis of the transport of the protein in soil, which indicated that the amount of proteins and degradation products decreased with increasing the distance from the microgranule. The maize plants derived from seeds were harvested after 33 days of cultivation and subjected to the mass analysis. The results of the analysis are shown in Figs. 10a-c. The largest mean mass of dry root equal to 2.83 g per g of the total plant was obtained for the maize plants fertilized with the microgranule supplemented with the whey protein isolate.

The whey protein supplement induced 37.2% increase in dry mass of the root compared to the plants fertilized without the protein supplements, whereas in case of the soy protein fortifier that increase was equal to 18.9%. The variance analysis performed indicated statistically significant influence both protein fortified fertilizers on dry mass of the root (Fig. 10a).

Additionally, dry mass of the aboveground part of the plant was also determined (Fig. 10b). The application of protein fortifiers also positively influenced on growth of that part of the plant. The largest mass of the aboveground part was achieved for the maize plant fertilized with the microgranule fortified with the whey protein isolate. Mean dry mass for the plants harvested from that source was 14.8% higher compared to those fertilized without the protein fortifiers. However, the variance analysis did not indicate statistically significant influence of addition of protein fortifiers on the amount of dry mass of the aboveground part of the plant in 33 days after sowing (BBCH14). Statistically significant differences were observed only between plants of the control variant (without the fertilizer) and other variants in which fertilizer was used.

The influence of the application of the fertilizer on the growth of root was also quantified as the mass ratio of the root to the aboveground part of the plant. The ratio is an indicator of availability of water and nutrients and their influence on the root growth. In this case, the best results were also achieved for the fertilizer fortified with the whey protein isolate. The plant-growth stimulating effect of that fortifier has been successfully verified under field conditions [26].

## Discussion

The analysis of the mobility of ions from the microgranule matrix in water environments revealed that the sulfate sodium and potassium ions were released from the matrix in majority. It was shown that also a small amount of phosphate ions contained in calcined bones and calcium ions was mobile. The analysis indicated that the microgranule was a source of hydroxyl ions coming from calcined bones. This caused a mild alkalization of microgranule environment (Fig. 2).

The ions contained in the microgranule matrix that were mobile in water environment were found to be also mobile in soil (Fig. 4); therefore, they could be used as a macronutrient for plants (e.g. potassium ions, small amount of phosphate and calcium ions). The proteins contained in the microgranule diffused through the solid matrix slowly - they did not practically reach the distance above 3 cm from the microgranule within the time interval investigated (Figs. 1, 5 and 9). Diffusion of proteins in soil was accompanied with their degradation, which was probably induced by microorganisms present in soil. A moderately alkaline pH environment of inside microgranule is expected to enhance the degradation process. The kinetics of the protein degradation in soil was also slow. The $\mathrm{N}{\mathrm{H}}_4^{+}$ ions, which were products of the protein degradation, were released from the microgranule after 72–168 h. This delay can act as positive stimulator of seed germination which starts in a few days after seeding. The degradation was the fastest for the whey protein, slower for the soy protein and very slow for the white egg protein from egg powder. In the latter case the degradation process was probably inhibited by poor solubility of egg powder in aqueous environment. The reported trends concerning diffusion and degradation of the protein were valid for both model soil systems investigated, i.e., composed of sand, as well as sand and peat (soil I and soil II). The protein concentration in soil II in the neighborhood of the microgranule was lower compared to soil I (Fig. 1 and Fig. 9), which might be explained by diffusion hindrances and, possibly, an enhancement of protein degradation in organic matter-reach soil. In all cases the diffusion path was short, therefore the microgranule should be placed just below the plant seed.

The results of the pot experiments revealed positive influence of the protein fortifiers on the root density. The best results were achieved for the whey-protein fortifier. A positive influence was also observed for microgranules fortified with the soy-protein. This proved that byproducts or waste of food industry, such as milk serum and soy, might directly be used to fortify fertilizers without previous treatments. The use of egg white powder might be possible after pretreatment by hydrolyzation, which however would involve additional costs.

## Conclusions

A microgranule fertilizer fortified by proteins was developed for local soil fertilization. The matrix of the granule consisted of calcined bone provided from food industry. Cheap products, which are byproducts or waste of food industry, such as milk serum and soy, were a source of proteins that could be easily degraded in soil without previous chemical or enzymatic pretreatments. The protein degradation induces formation of ammonium ions which may be utilized by plants as a nitrogen source. The maximum of the protein concentration and $\mathrm{N}{\mathrm{H}}_4^{+}$ ions is reached at a very close distance from the microgranule, therefore, the fertilizer microgranule should be placed in a direct vicinity to the plant seed. The microgranule matrix was also a source of ionic micronutrients: potassium, sulfate ions due to the presence of calcined bones, which were obtained as a post-production waste, a small amount of phosphate ions was also released.

Though local fertilization combined with seed sowing introduces a small amount of fertilizer into the soil, the site-location of the microgranules causes the fertilizer components to be available for plants at the right time and concentration. In this study, the tested dose of the fertilizer of 30 kg ha^− 1^ (0.33 g pot^− 1^) introduced 0.33 g of fertilizer into the soil per one maize plant. The amount of N, P, K and S delivered with that dose into the soil was very small in relation to germinating maize grain, whose mass was 0.264 g (mass from thousand seeds = 264 g), however it was effective enough to have an impact on the intensive growth and development of the emerging root system. The effect was confirmed by the results of the performed experiments, which revealed statistically significant positive influence both whey and soy-protein fortified fertilizers on dry mass of the root. However, the best results were achieved when the whey protein fortifier was used. The protein from egg white powder did not degrade in soil within the investigated time interval.

## Methods

### Materials

Three different sources of proteins were used as the fortifiers: whey protein isolate from milk serum (purchased form Olimp Labs), soy protein isolate and chicken egg white powder (both purchased from Bulk Powders). The composition of the protein material is presented in Table 2.

**Table 2:** Main components of the protein fortifiers

**Whey protein isolate % w/w**				
Protein	Carbohydrate	Fibre	Fat	Salt
87.0	1	0	0.6	0.85
**Soy protein isolate % w/w**				
Protein	Carbohydrate	Fibre	Fat	Salt
92.0	< 1 g	0	3.3	1.67
**Chicken egg white powder % w/w**				
Protein	Carbohydrate	Fibre	Fat	Salt
82.2	4.46	0	0.3	3.12

### Preparation of microgranule

The matrix of the microgranule was composed of calcined bones, which were obtained after incineration and grounding animal bones delivered from a slaughterhouse. The element composition of the matrix was as follows: P 137.0, Na 4.0, K 30.0, Mg 145.3, Ca 474.4 [mg g^− 1^], N 3.2 [mg g^− 1^], and Fe 1.0, Mn 6.5, Zn 0.8, Cu 3.2 [μg g^− 1^]. The fortified microgranule contained 2% w/w of the protein material.

The ingredients of the matrix were mixed in a V-type flow mixer for 2 h with the stirring velocity 10 rpm. The obtained mixture was granulated periodically using a pan granulator. Water was added as the binding liquid in the amount of about 18% w/w, the grain size varied within the range 1–3 mm. The method of production is protected by a patent application [27].

### Characteristics of the soil systems

Two standardized soil systems were used, i.e., the model soil I composed of dried sand with particle size 0.1–0.5 mm (provided by PPKiUG Kruszgeo SA, Poland), and the model soil II composed of a mixture of sand and a peat with pH_H2O_ 5.5–6.5 (provided by Planta Sp. z o.o., Poland) in proportion (7:1 w:w) homogenized under “dry” conditions.

Characteristics of soil I: pH_H2O_ = 6.26, low organic matter content (< 1% w/w), low content of macroelements P, K, Mg: P_2_O_5_- 4.58 mg, K_2_O - 2.19 mg, and Mg - 1.83 mg per 100 g of soil.

Characteristics of soil II: pH_H2O_ = 6.12, organic matter content 10.78% w/w, content of macroelements P, K, Mg: P_2_O_5_ - 11.2 mg, K_2_O - 4.39 mg, Mg - 29.66 mg per 100 g of soil.

Both soil I and soil II were mixed with water in the amount of 50% of the water holding capacity; for soil I it was 20% w/w, for soil II – 40% w/w.

### Measurement of the concentration of ionic species

The concentration of ions was determined using an ionic chromatography system Dionex 5000^+^ equipped with a conductometric detector. To determine the concentration of cations, a Dionex AS9 HC column with dimensions 250 × 4 mm and a guard column Dionex AG 50 × 4 mm were used. The measurement conditions were as follows: aqueous solutions of 18 mM Na_2_CO_3_ as the eluent, the flow rate 1 mL min^− 1^, the oven temperature 30 °C, a suppressor ASRS 4 mm, 45 mA current, the injection volume 25 μL, the detector was termostated at 35 °C.

To determine the concentration of anions, a Dionex CS 16 column with dimensions 250 × 5 mm and a guard column Dionex CG16 50 × 5 mm were used. The measurement conditions were as follows: aqueous solutions of 30 mM methanesulfonic acid as the eluent, the flow rate 1.4 mL min^− 1^, the oven temperature 40 °C, a suppressor CSRS 4 mm, 123 mA current, the injection volume 5 μL, the detector was termostated at 40 °C.

### Size exclusion (SEC) analysis

The changes in molecular weight of the protein fortifiers with time were analyzed using a chromatographic system Hitachi (Merck) with a UV detector and a data station. The system was equipped with a SEC-HPLC column TSKgel SuperSW3000 (TOSOH) with dimensions 300 × 4.5 mm and a guard column 3 cm × 4.5 mm, the bed particle diameter was 4 μm. The measurement conditions were: phosphate buffer pH 7 as the eluent, the flow rate 0.2 mL min^− 1^, the temperature for the measurement 22 °C, the injection volume 20 μL. Chromatograms were recorded by the detector at wavelength 280 nm.

### Measurement of mobility of microgranule ions in water

A 0.5 (±0.005) g microgranule was placed into a glass vessel and covered with 6 mL deionized water. The solution was stirred by a magnetic stirrer for 24 h at room temperature. Next, samples of supernatant were withdrawn and subjected to the ion concentration analysis. The experiments run in triplicate using always a fresh microgranule.

To determine the mobility of hydroxyl group from the matrix in aqueous environment, a 0.2 g microgranule was contacted with 5.0 mL deionized water. The solution was stirred for 30 min at room temperature. pH of the supernatant was measured using a pH electrode (Mettler Toledo InLab Ultra-Micro pH).

### Determination of transport of the microgranule components through soil

Seven identical 15 mL cylinders with a piston and a metric scale on the side-wall were used for each experimental run. A microgranule (0.5 g mass) fortified with the protein material was placed onto the bottom of each cylinder. Next, the cylinders were filled with soil I or soil II up to 4.5 cm height, sealed with a parafilm, and fixed vertically.

The cylinders were subsequently emptied at seven-time intervals, i.e., after: 1, 6, 24, 48, 72, 120, 168 h. At each time interval, 5 mL soil layers were taken at three different distances from the microgranule: 0–1.5 cm (soil layer 1), 1.5–3.0 cm (layer 2), 3.0–4.5 cm (layer 3), 4.5–6 (layer 4). Next, each soil sample was placed into a glass vial and mixed with 6 mL of a 0.1 M NaCl solution. NaCl salt was added to increase the ionic strength of the solution, which improved solubility of proteins and enhanced efficiency of their extraction from the matrix. The content of the vials was stirred using vortex for approx. 30 s, and then for 10 min using a magnetic stirrer to homogenize the mixture. The samples were allowed to settle. The supernatant was then filtered through a syringe filter and subjected to the composition analysis. The concentration of ions in samples was determined using ion chromatography, the change in molecular weight of the sample components was analyzed using SEC-HPLC, and the concentration of protein was determined by the Lowry method. The experiment was performed for microgranules supplemented with the protein fortifiers (milk serum isolate, soy protein isolate, and egg white powder) as well as without the protein additive.

Additionally, blank samples of soil I and II were subjected to extraction. The extracts of soil samples were analyzed in the same way as those contacted with the microgranules.

### Pot experiments

The pot experiments were performed in a cultivation room in pots with a capacity of 3 kg*.* The experiments were accomplished with five replicates for each of variants in completely randomized design. Four variants were used in the tests:

P0 - control variant (without the fertilizer);

P1 - fertilized by a microgranule matrix without the protein fortifier;

P2 - fertilized by a microgranule fortified with the whey protein isolate;

P3 - fertilized by a microgranule fortified with the soy protein isolate.

In the pot experiments, a qualified sowing material of maize seeds of the variety 5 EH Farmgigant A6R4131, RWA Raiffeisen Ware Austria AG Betriebsstaette Lannach (FAO 250) (mass of thousand seeds = 264 g) was used, which was purchased form FarmSaat AG. A single seed was planted at a depth of 4 cm. Microgranules with a diameter 2–3 mm were placed 2 cm below the maize seed in a dose of 0.33 g pot^− 1^ (with a plant density of 90,000 plants ha^− 1^, it corresponds to the fertilizer dose of 30 kg of fertilizer per ha). The model soil II (sand and peat) was used for the tests with a constant humidity of 50% of the maximum water-holding capacity (WHC), the same as that used in the mass transport measurements. The structure of natural soil is strongly heterogenous, therefore it can be expected that the diffusion rate of proteins and their degradation products might be dependent on the environment. In this study, to ensure reproducibility of the experiments, the artificial soil (i.e., soil II) was used that was characterized by a high structure homogeneity.

The plants were harvested 33 days after sowing (BBCH14). The root and the aboveground parts of the plants were washed and dried to determine dry mass. The results were statistically evaluated by use of a Statistica 10.1 software based on the variance analysis with a significance level of 0.05. The measurements and statistical evaluation were performed by dr Natalia Matłok.

## Supplementary information

**Additional file 1: **Supplementary materials to the manuscript including five figures: **Figure S1** - mean concentration of K^+^ ion with time in soil I; **Figures S2-S5** depict the progress of diffusion of proteins and ions in soil I for the fertilizer fortified with egg white powder.

## Data Availability

The datasets used and/or analyzed during the current study available from the corresponding author on reasonable request.

## Abbreviations

A: Absorbance
BBCH14: Amount of dry mass of the aboveground part of the plant in 33 days after sowing
P0: Control variant (without the fertilizer)
P1: Fertilized by a microgranule matrix without the protein fortifier
P2: Fertilized by a microgranule fortified with the whey protein isolate
P3: Fertilized by a microgranule fortified with the soy protein isolate
rpm: Revolutions per minute
SEC-HPLC: Size exclusion high performance liquid chromatography
*t*: Time
% w/w: Percent by mass
*x*_*p*_: Mass fraction of protein
*x*_*ion*_: Mass fraction of inorganic ion
