# Supplementary material for: Mechanism of nutrition activity of a microgranule fertilizer fortified with proteins
Source: BMC Plant Biol. 2020 Mar 24;20:126. doi: 10.1186/s12870-020-02340-4 (PMC7092569; doi:10.1186/s12870-020-02340-4)
Supplement: Supplementary file 1 — Additional file 1: Supplementary materials to the manuscript including five figures: Figure S1 - mean concentration of K+ ion with time in soil I; Figures S2-S5 depict the progress of diffusion of proteins and ions in soil I for the fertilizer fortified with egg white powder. [file 12870_2020_2340_MOESM1_ESM.docx]

# Supplementary materials


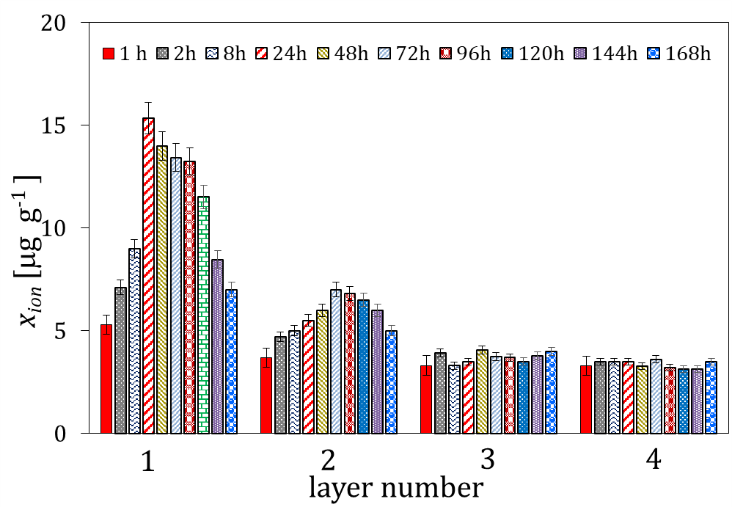


Fig. S1. Mean concentration of K^+^ ion with time in the extracts from soil I layers (soil layer 1: 0-1.6 cm, layer 2: 1.6-3.2 cm, layer 3: 3.2-4.8 cm, layer 4: 4.8-6.0 cm) acquired at different distances from the microgranule.


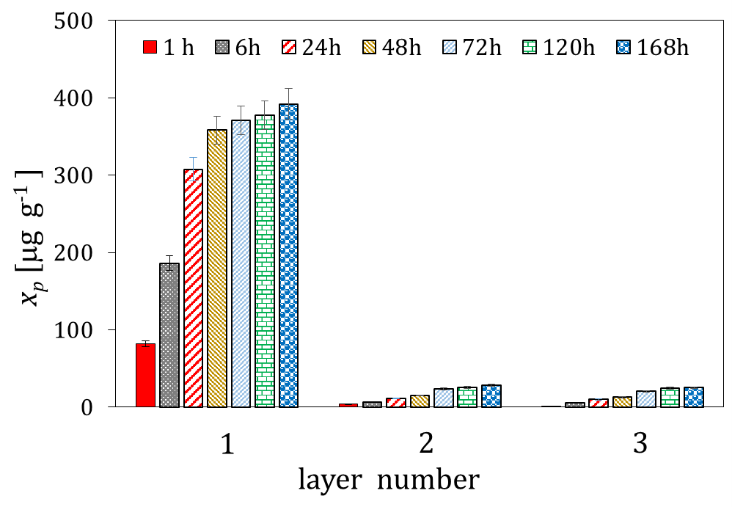


Fig. S2. Changes in the mean protein concentration with time in the extracts from the soil layers acquired at different distances from the microgranule fortified with the egg white protein powder.


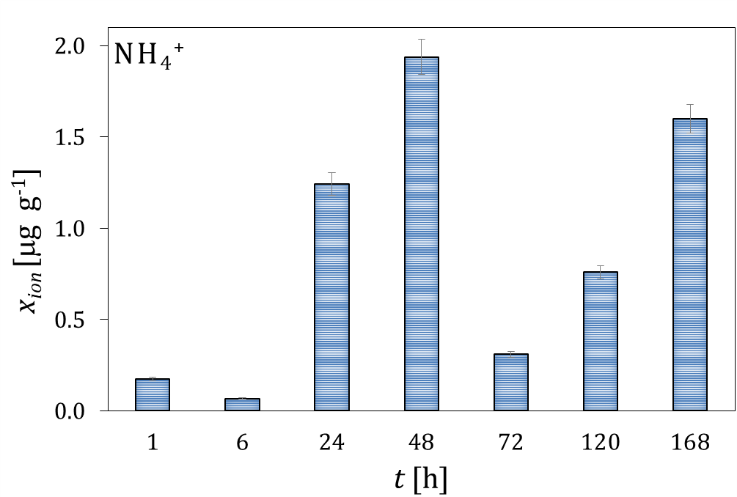


Fig. S3. Changes in the man excess concentration of $NH_{4}^{+}$ ions with time in the extracts from the soil layer 1 surrounding the microgranule fortified with the egg white powder.

Fig. S4. SEC-HPLC chromatograms for a standard solution and the extracts of soil I. A) Soy protein isolate as a standard and the extract from the soil layer 1 acquired after 72 h; B) extracts from the soil layer 1 acquired at different time intervals.


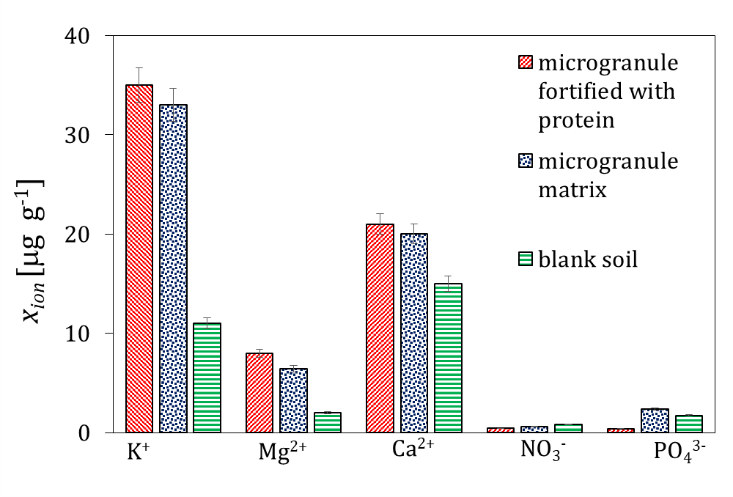


Fig. S5. Mean concentration of ions in the extracts from the soil layer **1** acquired after 168 h; blank sample without the contact with the microgranule, sample after the contact with the microgranule free of the protein, and with the microgranule fortified with the egg white powder.
